# Role and Effects of Hippotherapy in the Treatment of Children with Cerebral Palsy: A Systematic Review of the Literature

**DOI:** 10.3390/jcm10122589

**Published:** 2021-06-11

**Authors:** María José Menor-Rodríguez, Mar Sevilla Martín, Juan Carlos Sánchez-García, María Montiel-Troya, Jonathan Cortés-Martín, Raquel Rodríguez-Blanque

**Affiliations:** 1 Research Group CTS367, E.U.E. Campus Ourense, University of Vigo, 32616 Ourense, Spain; maria.jose.menor.rodriguez@sergas.es; 2 Area Sanitaria Ourense-Verín-Barco de Valdeorras, 32800 Ourense, Spain; mar.sevilla.martin@sergas.es; 3 Research Group CTS1068, JMADOC, School of Nursing, Faculty of Health Sciences, University of Granada, 18071 Granada, Spain; 4 Research Group CTS1068, School of Nursing Ceuta Campus, Faculty of Health Sciences, University of Granada, 51001 Ceuta, Spain; 5 Research Group CTS1068, Hospital Universitario Virgen de las Nieves, 18014 Granada, Spain; 6 Research Group CTS1068, Distrito Sanitario Granada-Metropolitano, 18013 Granada, Spain; raquel.rodriguez.blanque.sspa@juntadeandalucia.es

**Keywords:** hippotherapy, equine assisted therapy, children, cerebral palsy

## Abstract

Cerebral palsy is described as a group of permanent neuromotor-type disorders caused by non-progressive injuries in the developmental stages of the central nervous system, and which have serious repercussions on the quality of life of affected children due to the physical and psychological damage it entails for them. Today, it is the leading cause of physical disability in childhood. Since there is no cure for this disorder, treatment is based on the improvement of symptoms, which is not always achieved through conventional therapies. For this reason, the need arises to investigate other alternative therapies, such as hippotherapy, to determine the main effects of hippotherapy as a rehabilitation therapy in children with cerebral palsy. The review was performed in accordance with the criteria of the Preferred Reporting Items for Systematic Reviews and Meta-Analyses (PRISMA) protocol and was registered under the number CRD42021233003. The databases used were PubMed, Dialnet and the web browser Google Scholar. After applying the inclusion criteria, we included 11 articles. As a conclusion, we found that hippotherapy provides benefits at physical, psychological, cognitive and social levels in children with cerebral palsy, and thus it should be considered as a complementary rehabilitation therapy to conventional treatments.

## 1. Introduction

Cerebral palsy (CP) is a group of permanent neuromotor-type disorders that are caused by nonprogressive disturbances during the prenatal, perinatal or postnatal (2–3 years) stages of development of the central nervous system [1,2]. Children affected by this syndrome present with postural and motor disability that entails limitations in their functional abilities and physical activity, leading to impaired physical, psychological and social development [3,4]. The prevalence of CP in developed countries is between 2 and 2.5 per 1000 live births. It is the main cause of physical disability in childhood [5].

There are many factors that can lead to CP. Depending on the stage at which the disturbances take place, they can be prenatal, perinatal or postnatal, although perinatal is the most common and represents 60% of cases [2,5,6]. It is of great importance to understand these factors in order to prevent the development of CP and allow for early detection. This will ensure a closer surveillance that will enable a better quality of life for these children from the start and avoid a worse progression of the disorder. The diagnosis of CP is usually reached after the neonatal period when the first distinctive signs of the disorder are detected, although earlier on, certain neurological signs may arouse suspicion [2].

Among the most noticeable early signs are abnormal muscle tone, reflexes and posture and developmental delays. Other clinical signs and symptoms include sensory-perceptual alterations (hearing problems, vestibular or balance disorders, clumsy hands and fingers), impaired coordination, muscle spasms, speech impairment and learning disabilities (although in some cases, these functions may be preserved), spasticity and tremors, involuntary movements, abnormal posture and muscle tone, seizures or mental impairment (difficulty concentrating and developing ideas, anxiety) [7]. CP has a highly variable presentation; therefore, it can be classified according to three different criteria in order to facilitate comprehension and treatment: according to the part of the body that is affected, according to motor function or according to severity [5,7,8].

There are different tools for the evaluation of CP. The most commonly used scales include the Gross Motor Function Classification System (GMFC system) [9], the Ashworth scale [10], between measurement and spasticity and the Berg balance scale [11] to assess balance abilities.

As no curative treatment exists for cerebral palsy, treatment usually involves specific physiotherapy techniques and psychological therapies that aim to improve the child’s symptoms and quality of life [8].

There are several types of treatment. Among the most conventional treatments are “Bobath therapy” and “Vojta therapy”, focused mainly on posture and movement disorders, attempting to normalize them as much as possible, as well as different early stimulation therapies that work at the central nervous system level in a sensory way [4,12].

In the past few years, another type of therapy, known as “complementary” therapy, has started to gain visibility. Among such therapies is hippotherapy or therapy with horses, supported by its many benefits at both physical and mental levels in different diseases, including CP [13].

The therapeutic use of horses goes back to ancient times. In the fifth-century B.C., Hippocrates described the usefulness of horse riding in the treatment of different ailments such as insomnia or in the improvement of muscle tone in his writings “On Regimen” [14,15]. Hippotherapy is a kinetic rehabilitation therapy that has effects on physical, mental and emotional aspects [16]. It is directed at patients with neurological/neuromotor alterations of degenerative or traumatic origin [12]. It is based on the utilization of the horse’s movements, which provide sensitive and motor stimuli, for the treatment of different disorders in which the patient is able to perform no or little movement on the horse. This therapy contributes to functional and psychological rehabilitation and improves basic abilities and social adaptation [17]. Since the person is not able to execute any action on the horse, it is the horse who acts upon the patient, and in so doing, becomes a co-therapist and take on a major role in the therapy. The horse is guided by an expert in equine therapy who directs it at the most adequate rhythm for each patient (walk or trot, but never gallop) [5,14].

Hippotherapy has three known therapeutic principles. Firstly, the transmission of body heat. The horse’s body temperature is 38 °C and may reach 38.8 °C during exercise [5]. This temperature, higher that the human body’s, is useful for the extension and relaxation of muscles and ligaments and for the increase in sensory perception due to the tactile stimuli it generates [1,2].

Secondly, the transmission of rhythmic impulses. When walking, the horse sends around 90–110 rhythmic impulses or vibrations to the pelvic belt. These impulses are generated from the alternate elevation of the lumbar muscles and the horse’s back. Such elevations create an oscillating movement in the patient’s pelvis, so the patient must adapt and straighten the trunk. In doing so, balance, postural control and coordination are improved [2,5,18]. On a psychotherapeutic level, the impulses generate a feeling of being worthy, awakening in turn feelings of safety and protection that build up the patient’s self-confidence [1,2].

The third principle is the transmission of a three-dimensional locomotion pattern. This pattern is similar to human gait, where three different movements are generated: anteversion-retroversion, lateral-rotational displacement and descent-elevation [14]. This creates an increase in elasticity and flexibility of the pelvic ligaments. It also contributes to the stabilization and coordination of the trunk and head due to the automation of the rhythmic pattern of the horse. The similarities with the physiologic pattern of human gait enable its acquisition while remaining seated, with no need for the use of the lower extremities [2,18].

In order to achieve the most effective treatment possible, the nursing staff involved in such therapies must always be qualified and trained in the field of equine therapy, with a total understanding of the characteristics and behaviors of the horse and the possibilities it entails for healthcare [15,19,20].

CP remains the main cause of physical disability in childhood [17]; therefore, there is a need for research in new alternative methods that will complement conventional medical treatments in order to achieve the most effective improvement of symptoms possible.

In countries such as the United States of America, France or England, there have been noticeable results in the application of hippotherapy in the treatment of different disorders, possibly due to the existence of equine therapy federations and the requirement of professional qualifications [2,17].

Equine therapy does not have the expected relevance due to the high costs of this type of treatment, as well as the possible prejudices parents may have regarding the risks of treatment with animals due to fears of irreparable damage that they may cause their child.

These issues led to the development of this literature review to answer the following question: Does hippotherapy have a beneficial impact in the rehabilitation treatment of children diagnosed with CP?

The main objective was to determine the main benefits provided by hippotherapy as a rehabilitation technique in children with CP, and the specific objectives were to describe how hippotherapy influences gross motor function, balance and postural control, spasticity and muscle tone and to describe the psychological, cognitive and social effects of hippotherapy and its effects on dependency for activities of daily living.

## 2. Materials and Methods

The protocol of this review can be found online at: http://www.crd.york.ac.uk/PROSPERO/ (accessed on 12 April 2021) under the registration number CRD42021233003.

This review was carried out following the Preferred Reporting Items for Systematic Reviews and Meta-Analyses (PRISMA) protocol, which consists of a 27-item checklist of the most important sections of an original article as well as a flow diagram that depicts the flow of information through the different phases of a systematic review.

Before the elaboration of the literature review, we used different primary sources in an extensive documentation search on the study issue between December 2018 and March 2019.

### 2.1. Eligibility Criteria

The search strategy followed attempted to respond to the question formulated in the objectives: What are the main benefits of hippotherapy as a rehabilitation technique in children with CP? The PICO system was used as a guide (Population—therapies in children with CP; Intervention—hippotherapy; Comparison—there is no comparison group in this study; Outcomes—positive effects).

We included all the studies in which the patients were 18 years old or younger, of either sex, diagnosed with CP who had received treatment with hippotherapy with real horses. We excluded reviews and meta-analyses, studies that included patients with diagnoses other than CP and all studies on hippotherapy with horse simulators. The filters used were research articles, complete text, published between 2009 and December 2020, in English or Spanish.

Articles considered irrelevant for the purpose of this review were also excluded.

### 2.2. Information Sources and Search

The primary sources used were electronic databases such as PubMed, Dialnet and the online search engine Google Scholar. We checked the health-related descriptor pages DeCS (Descriptores en Ciencias de la Salud) and MeSH (Medical Subject Headings) and determined the search parameters for data collection on the chosen databases. The descriptors we used were hippotherapy, equine-assisted therapy, children and cerebral palsy.

All descriptors were combined using the Boolean operators “AND” and “OR” (see search strategy in Supplementary Table S1).

### 2.3. Selection Process

After searching the different databases, we proceeded to eliminate duplicate documents. Following the removal of duplicates, we screened all the retrieved articles by title and abstracts to identify those that fit the inclusion criteria. Next, we independently read each article, focusing on its methodology to verify whether it complied with our eligibility criteria, and all articles that did not were discarded.

Three independent reviewers completed this phase of the selection process. Any disagreements were discussed with the study supervisor and resolved via a consensus-based discussion.

### 2.4. Data Collection Process

All articles found were transferred to the web application Mendeley using the Mendeley web importer tool.

After importing all the articles to the Mendeley website, we organized them by folders according to the database from which each article had been retrieved and proceeded to eliminate all duplicates, thereby achieving the definitive list for our study.

### 2.5. Study Selection

The study selection process was initially based on a screening of the titles and abstracts of all the retrieved articles in order to select those which fulfilled the study objective. Next, we comprehensively read each article, focusing on its methodology to verify whether they complied with our eligibility criteria. We only included the documents that met the inclusion criteria related to the research question established for this review. This process was carried out by two researchers and in cases where any disagreements arose, the arbitration of the study supervisor was sought.

### 2.6. Data Items

We retrieved the following data: author, title, year of publication, methodological quality of the studies, type of article, objectives of the article, intervention performed and its phases, instruments used in the measurement of variables and the obtained conclusions.

### 2.7. Risk of Bias in the Individual Studies

For the methodological evaluation of the eleven articles selected for this study, we analyzed the design, methods and type of study of each article in order to select the most specific methodological evaluation scale for each case [21]. Of the eleven articles, seven had an experimental design (randomized control trials, RCT) and four were case reports.

The quality of the studies was evaluated using the Jadad scale or the Oxford quality scoring system [22]. This procedure is used to independently assess the methodological quality of a clinical trial. The Oxford scale only considers aspects related to possible biases regarding randomization, blinding that makes patients and researchers unaware of treatment allocation (known as double blind) and the description of those lost to follow up. It is a simple questionnaire that is validated and quick to apply.

This questionnaire allocates a score on a scale of 0 to 5 points, where the higher the score the better the methodological quality of the clinical trial.

Points are given as follows: study described as randomized, 1 point; adequate randomization method, 1 additional point; inadequate randomization method, take away 1 point; patient blinded to the intervention (patient blinding is presumed when the intervention of the control group was indistinguishable from the treatment group), 1 point; evaluator blinded to the intervention; 1 point; description of withdrawals and dropouts, 1 point.

A 5-point clinical trial is considered “rigorous”. A clinical trial is considered of poor quality if it scores below 3 points.

The reliability of this scale has a Kappa variation between good and excellent (k = 0.61 to k = 0.88) and the CCI from bad to excellent (0.39–0.91) [21].

The Charlson Comorbidity Index (CCI) is a system for evaluating life expectancy at ten years, depending on the age at which it is evaluated and the comorbidities of the subject, relating long-term mortality with the patient’s comorbidity. In general, absence of comorbidity is considered 0–1 point, low comorbidity 2 points and high comorbidity >3 points [21].

The articles with a case report design were evaluated using the SCED scale (Rating Scale for Single Participants Designs) [23]. This scale was constructed using 11 items, ten of which are used to assess methodological quality and one is for the use of statistical analysis. The SCED scale presents an excellent inter-evaluator reliability for the total score. Item reliability was fair to excellent for consensus ratings between pairs of raters.

## 3. Results

**Figure 1:**
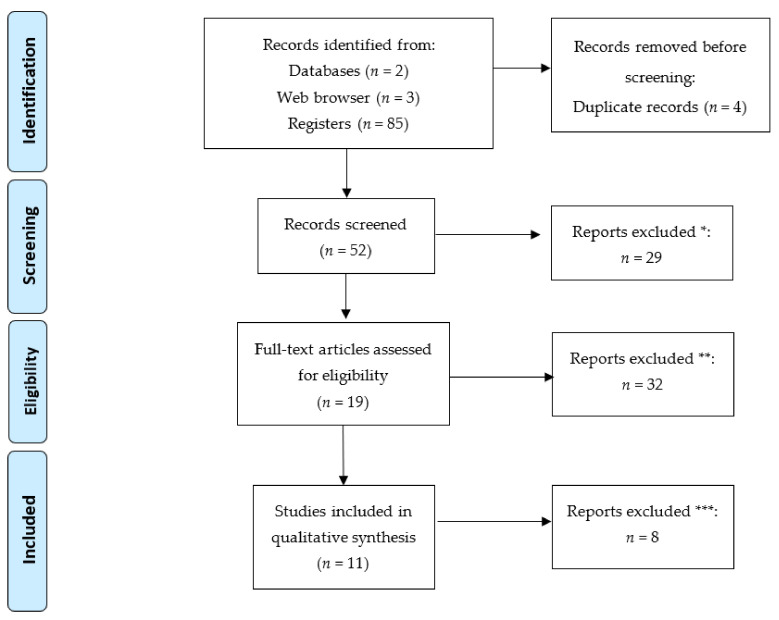
Flow diagram. * Studies excluded after reading title and abstract. ** Studies on other animals as well as horses; studies on other disorders. *** Studies excluded after reading the complete article.

Figure 1 shows the flow diagram of the articles assessed during the production of this systematic review.

### Study Characteristics

As we previously mentioned in the methods section, we proceeded to analyze the selected studies in order to assess the scientific quality of each one by using the Oxford scale for RCT and the SCED scale in case reports.

The studies that obtained the highest scores in the methodological evaluation of the seven RCT were the studies by McGibbon et al. [24] and Lucena-Antón et al. [25] at 5 points and an excellent qualification; the rest of the articles have a score of 3 points, so none of them can be considered of poor quality (see Supplementary Materials, Table S2).

Regarding the single case reports, the studies by Fernández Gutiérrez et al. [26] and Rodríguez Laiseca et al. [27] obtained scores of 11/11, the study by Fourmantin [8] obtained 10/11 and the one by Paternina [28] scored 8/11 (see Supplementary Materials, Table S3).

Finally, eleven studies were selected and included in this review (see Table 1).

## 4. Discussion

The main objective of this literature review was to determine the main effects of hippotherapy as a rehabilitation therapy in children with CP.

It would appear, according to the 11 selected articles, that hippotherapy has, to a greater or a lesser extent, beneficial effects on different aspects (physical, psychological, cognitive and social) in children with CP.

In general, the assessment of the effects obtained from the treatment was carried out immediately after applying equine therapy over a certain period (from a couple of weeks up to a year). Only McGibbon et al. [24] and Rodríguez Laiseca and Lerma Castaño [27] evaluated after a period of therapeutic inactivity, yielding conflicting results. In the first of the mentioned studies, researchers found that hippotherapy is effective whilst its application is continuous; however, in the second study, results showed that the effects of such treatment persist beyond discontinuation of therapy sessions.

Matusiak-Wieczorek et al. [29] and Lucena-Antón et al. [25] compared the action of rehabilitation with only physiotherapy and that of a combination of physio and hippotherapy.

On the other hand, McGibbon et al. [24] also compared the difference between treatment with hippotherapy and conventional physiotherapy on a stationary barrel, for an improvement in adductor muscle asymmetry. The results favored hippotherapy for greater improvements.

Regarding the variables considered in the different studies:

### 4.1. Gross Motor Function

Three studies evaluated the effects of hippotherapy on this variable and all agreed on an improvement after treatment.

Both Paternina [28] and Villegas Guerrero [7] found that there is an increase in movement-related functions, allowing the children to initiate and, in some cases, complete postural changes autonomously, as well as a decrease in involuntary movements than limit motor coordination when performing activities.

McGibbon et al. [24] also found that the improvements in gross motor function last up to 12 months after treatment.

### 4.2. Balance and Posture Control

Five studies assessed the variables of balance and posture.

Fernández-Gutiérrez et al. [26] concluded that after the application of equine therapy there are clear improvements in body weight distribution and the location of center of gravity, leading to a greater postural stability.

According to Matusiak-Wieczorek et al. [29] and Paternina [28], a better stability and control of the head, neck, trunk and extremities (more so upper than lower) also play a role in postural improvement.

On the other hand, such postural stability leads to an important increase in balance. However, results are conflicting in the degree of improvement, as Fourmantin [8] found that the improvement in balance was progressive over the duration of therapy, whilst Delgado Fernández and Sánchez Gómez [32] reported that the true difference was only detected after 6 months of treatment.

Paternina [28] also found that achieving a certain balance and functional posture favors children’s mobility and autonomy and therefore improves their quality of life.

### 4.3. Spasticity and Muscle Tone

Six studies considered the effects of hippotherapy on spasticity and muscle tone.

We found that they all focused on the increase in size and muscle tone, essential factors in reducing spasticity.

Fourmantin [8], Rodríguez Laiseca and Lerma Castaño [27] and Jami Vargas et al. [30] observed that such reduction in spasticity happens on a general level, but the second study highlighted three specific muscle groups: elbow flexors, plantar flexors and hip extensors. In that same study, the authors indicated the necessity of continuous application of hippotherapy for the maintenance of these effects.

The three remaining studies, which include those of McGibbon et al. [24], Lucena-Antón [25] and Reyes Domínguez [31], focused on a reduction in the spasticity of hip adductor muscles. Their results were also positive due to an improvement in the range of movement and muscle symmetry, which facilitate walking.

### 4.4. Psychological, Cognitive and Social Effects

McGibbon et al. [24], Paternina [28] and Jami Vargas et al. [30] also analyzed psychological, cognitive and social variables in their studies. They all found an improvement in self-esteem and self-perception.

The second study observed that hippotherapy improved socialization of children with CP due to contact with the horse, and the third study emphasized the stimulation of concentration and attention over the course of therapy.

### 4.5. Dependency for Activities of Daily Living (ADL)

Finally, the article by Paternina [28] and the study by Delgado Fernández and Sánchez Gómez [32] included dependency for activities of daily living as a variable in their research. Both studies found that, to a lesser or greater extent, there was an improvement in the independent performance of activities of daily living after treatment with horses.

### 4.6. Limitations

As therapy with horses is currently still a rehabilitation discipline in full development, the number of articles published is limited. This fact may have in turn limited the possibilities of our review.

On the other hand, bearing in mind the aforementioned fact, we found that in the literature reviewed there is no uniformity in sample size or study duration, which may constitute a selection bias in our review.

## 5. Conclusions

This review of the literature shows that hippotherapy has positive effects on the health of children with CP. It is true that there are authors such as McGibbon et al. [24] and Rodríguez Laiseca and Lerma Castaño [27] who clarify this aspect in their studies, debating whether the efficacy of this therapy goes beyond its specific application or not. It should also be noted that there are discrepancies between the authors Fourmantin [8] and Delgado Fernández and Sánchez Gómez [32] on whether the improvement in balance was progressive during the duration of therapy or appeared months later. In any case, none of these authors deny the positive effects of hippotherapy on the health of children with CP, so it should probably be considered as a complementary rehabilitation therapy to conventional treatments.

The main variables that benefit from hippotherapy are gross motor skills, control of balance and posture, spasticity and muscle tone, dependency for activities of daily living and the psychological, cognitive and social spheres.

Due to the limited literature available on hippotherapy, there is a great need for more reviews on this topic, including studies with larger sample sizes and a common methodology regarding duration of therapy and study variables.

## Figures and Tables

**Table 1:** Most relevant results.

Authors	Description	Objetive	Intervention	Results
Fernández-Gutiérrez et al. (2015) [26]	Descriptive observational study.	They measured the effects of hippotherapy on the postural stability of an 8 years old girl diagnosed with CP with right hemiparesis and classified in levels I–II on the GMFCS scales of gross motor function.	Fourteen 14 hippotherapy sessions were performed, each of them once a week with 45 min duration.	Hippotherapy produces positive changes in the distribution of plantar support, the location of the center of gravity and postural stability, with an improvement greater than 25%.
Rodríguez Laiseca and Lerma Castaño (2015) [27]	Descriptive observational study.	They described the changes in muscle tone caused by hippotherapy in two children of two years of age with CP, both with symptoms classified in level V of the GMFCS.	Patients attended therapy with horses three times a week, for a total of 27 sessions of 20 min each.	Hippotherapy achieves immediate and medium-term effects on muscle tone. The results are positive when applying the treatment continuously, but they do not last over time, so there are no long-term effects.
Paternina (2013) [28]	Descriptive observational study.	They studied the influence of hippotherapy on different physical and psychological aspects of a 4-year old girl with CP.	The patient received 4-hour long hippotherapy sessions, twice a week for one month.	Hippotherapy improves the control of involuntary movements. It is possible to acquire a locomotion pattern allowing movement thanks to a greater balance and functional posture. Improved motor skills, achieving smoother and more controlled movements.
Matusiak-Wieczorek et al. (2016) [29]	Cohort study.	They evaluated the influence of hippotherapy on body balance in seating position in 39 children aged between 6 and 12, all of them classified in levels I and II of the GMFCS scale.	Patients were divided into two groups: 19 children were selected for the intervention group and 20 children constituted the control group. The intervention group attended hippotherapy sessions combined with physiotherapy rehabilitation, whilst the control group only attended the rehabilitation sessions. The therapy with horses took place once a week for 12 weeks, each session lasting 30 min.	Hippotherapy improves trunk control and the ability to maintain balance in a sitting position. There is greater control of the arms and hands, but the effects on the feet are less.
Fourmantin (2012) [8]	Descriptive observational study.	They aimed to improve developmental disorders in children with spastic cerebral palsy.	Patients participated in the intervention two or three times a week.	Hippotherapy achieves positive results in improving balance and stability, in posture and in the width of the angle of the joints. In addition, there is an increase in the confidence of patients in themselves.
Jami Vargas et al. (2016) [30]	Descriptive observational study.	They researched the benefits of hippotherapy on 13 children with CP (aged between 3 and 6 years old).	Patients attended therapy with horses three times a week for 10 weeks. The duration of each session was 30 min.	Hippotherapy has beneficial effects at the motor, cognitive and social levels, with the motor area being the one that obtains the best results. It is also possible to stimulate the capacity for concentration, attention and self-esteem during riding.
McGibbon et al. (2009) [24]	Cohort study.	Phase 1: they measured the immediate effects of hippotherapy in the symmetry of adductor muscle activity and their functional ability in 47 children (aged between 4 and 16 years old). Phase 2: they studied the long-term effects of hippotherapy in the symmetry of adductor muscle activity and their functional ability in 6 of the children previously selected for phase 1.	Phase 1: 10-minute treatment session before the evaluation of results. Phase 2: 30-minute hippotherapy sessions once a week for 12 weeks.	Hippotherapy produces improvements in the symmetry of the adductor muscles during ambulation, both in the short term and the long term. In addition, better results are observed in hippotherapy treatment compared to stationary barrel treatment.
Villegas Guerrero (2018) [7]	Descriptive observational study.	Application of hippotherapy in 15 children with cerebral palsy aged between 3 and 12 years old with the aim of improving their neuromusculoskeletal functions and those related to movement.	Twice a week during 8 weeks.	Hippotherapy leads to a significant improvement in movement functions in the items evaluated (turning, decubitus and sitting), showing progress at the level of gross motor function.
Reyes Domínguez (2013) [31]	Quasi-experimental study.	They researched the effect of equine therapy on the openness of the range of movement of hip adductor muscles in 21 children aged between 2 and 12 years.	10 once-weekly 30-minute treatment sessions.	Hippotherapy favors the openness of the range of movement of hip adductor muscles of the lower extremities, thus facilitating ambulation.
Delgado Fernández and Sánchez Gómez (2014) [32]	Quasi-experimental study.	To demonstrate the possible application of hippotherapy as a rehabilitation alternative in the treatment of CP in a group of 20 children aged between 8 and 11 years old.	The children attended 2-hour long horse assisted therapy sessions three times a week for a year.	Hippotherapy obtains positive results at the level of balance, making pedestrian walking possible over time. There is also a higher performance of the Basic Activities of Daily Living, achieving greater independence.
Lucena-Antón et al. (2018) [25]	Cohort study.	They assessed the effects of hippotherapy on muscular spasticity of 44 children of a mean age of 8.	Two groups: a control group of 22 children who received conventional therapy and an intervention group of another 22 children who received additional hippotherapy to the conventional therapy. This treatment plan was followed once a week for 12 weeks, in 45-minute sessions.	Hippotherapy improves short-term spasticity of the adductor muscles of the legs and the hip in children with CP who do not walk, by increasing muscle tone, balance and mobility of the hips.

CP, cerebral palsy; GMFCS, Gross Motor Function Classification System.

## Supplementary Materials

The following are available online at https://www.mdpi.com/article/10.3390/jcm10122589/s1, Table S1: Search strategy, Table S2: Results, methodological evaluation of RCT according to the PEDro scale, Table S3: Results, methodological evaluation of case studies according to SCED scale.
