# Supplementary material for: Role and Effects of Hippotherapy in the Treatment of Children with Cerebral Palsy: A Systematic Review of the Literature
Source: J Clin Med. 2021 Jun 11;10(12):2589. doi: 10.3390/jcm10122589 (PMC8230898; doi:10.3390/jcm10122589)
Supplement: Supplementary file 1 [file jcm-10-02589-s001.zip › jcm-1193252-supplementary.pdf]

**Table S1. Search strategy.**

| DATABASES AND SEARCH ENGINES | SEARCH STRATEGY | ITEMS |
|---|---|---|
| **PUBMED** | (("equine assisted therapy"[MeSH Terms] OR ("equine assisted"[All Fields] AND "therapy"[All Fields]) OR "equine assisted therapy"[All Fields] OR ("equine"[All Fields] AND "assisted"[All Fields] AND "therapy"[All Fields]) OR "equine assisted therapy"[All Fields]) AND "cerebral palsy"[All Fields] AND ("child"[MeSH Terms] OR "child"[All Fields] OR "children"[All Fields] OR "child s"[All Fields] OR "children s"[All Fields] OR "childrens"[All Fields] OR "childs"[All Fields])) AND ((clinicaltrial[Filter] OR randomizedcontrolledtrial[Filter] OR review[Filter]) AND (fft[Filter]) AND (english[Filter] OR spanish[Filter]) AND (2009:2020[pdat])) | 19 |
| **DIALNET** | hippotherapy AND cerebral AND palsy. Filters: children, Publication date: 2009-2020. | 16 |
| **SCHOOLAR GOOGLE** | "equine assisted therapy", "cerebral palsy", children. Filter: All, English or Spanish, Not Meta-Analysis, Not Systematic Review. Publication date: 2009-2020. | 50 |

*Source: self made*

**Table S2. Results. methodological evaluation of RCT according to the Oxford scale.**
*Source: self made*

| AUTHOR | McGibbon et al. | Lucena-Antón et al. | Matusiak-Wieczorek et al. | Jami Vargas et al. | Delgado Fernández et al. | Reyes Domínguez et al. | Villegas Guerrero et al. |
|---|---|---|---|---|---|---|---|
| **Described as randomized*** | 1 | 1 | 0 | 0 | 0 | 0 | 0 |
| Described as doble-blind* | 1 | 1 | 0 | 0 | 0 | 0 | 0 |
| Described of withdravals* | 1 | 1 | 1 | 1 | 1 | 1 | 1 |
| Randomization method described and appropriate** | 1 | 1 | 1 | 1 | 1 | 1 | 1 |
| Double-blinding method described and appropriate** | 1 | 1 | 1 | 1 | 1 | 1 | 1 |
| **TOTAL SCORE** | 5 | 5 | 3 | 3 | 3 | 3 | 3 |

* A study receives a score of 1 for "yes" and 0 for "no"

** A study receives a score of 0 if no description is given, 1 if the method is described and appropriate, and -1 if the method es described but inappropriate

# The word "double-blind" was not used by the authors. However, according to the description of the blinding of the investigator, investigational site staff, and participants, one point was given for "described as double-blind".

**Table S3. Results methodological evaluation of case studies according to SCED scale.**

| AUTHOR | The medical history has been specified. It should include age, sex, etiology, and severity. | Target behaviors. accurate and repeatable measurements that are operationally defined. Specify measure of target behavior. | Design 1: 3 phases. Study must be ABA or multiple baseline. | Design 2: baseline (pre-treatment phase). Sufficient sampling was carried out. | Design 3: Treatment phase. Sufficient sampling was carried out. | Design 4: Data record. raw data points were reported. | Observer bias: inter-rater reliability was established by at least one measure of objective behavior. | The independence of the evaluators. | Statistical analysis. | Replication: either through themes, therapists, or adjustments. | Evidence for generalization. | TOTAL SCORE |
|---|---|---|---|---|---|---|---|---|---|---|---|---|
| **Fernández-Gutierrez et al.** | YES | YES | YES | YES | YES | YES | YES | YES | YES | YES | YES | **11** |
| **Rodriguez Laiseca et al.** | YES | YES | YES | YES | YES | YES | YES | YES | YES | YES | YES | **11** |
| **Fourmantin et al.** | YES | YES | YES | YES | YES | YES | YES | YES | YES | NO | YES | **10** |
| **Paternina et al.** | YES | YES | YES | YES | YES | YES | NO | NO | YES | NO | YES | **8** |

*Source: self made*
